# Recent Small-Molecule Inhibitors of the p53–MDM2 Protein–Protein Interaction

**DOI:** 10.3390/molecules25051211

**Published:** 2020-03-07

**Authors:** Anastasia Beloglazkina, Nikolai Zyk, Alexander Majouga, Elena Beloglazkina

**Affiliations:** M.V. Lomonosov Moscow State University, Leninskie gory, 1-3, 119991 Moscow, Russia; anastas-beloglaz@mail.ru (A.B.); zyk@org.chem.msu.ru (N.Z.); alexander.majouga@gmail.com (A.M.)

**Keywords:** p53–MDM2 inhibitors, anticancer drugs, cytotoxicity

## Abstract

This review presents the last decade of studies on the synthesis of various types of small-molecule inhibitors of the p53– Mouse double minute 2 homolog (MDM2) protein–protein interaction. The main focus is placed on synthetic approaches to such molecules, their cytotoxicity, and MDM2 binding characteristics.

## 1. Introduction

In recent years, a number of reviews on p53–MDM2 interaction inhibitors have been published [1,2,3,4,5]. These reviews indicate continuing research interest on the topic. We note, however, that none of the reviews referenced above cover all aspects of p53–MDM2 interaction inhibition: biological, chemical, and medical. Sanz et al. [1] described the biomedical aspects of the p53–MDM2 interaction inhibitors, Nayak et al. [2] focused on the study of the structural aspects of p53–MDM2 interaction inhibitors, and the review by Estrada-Ortiz and co-authors [3] is devoted to an analysis of the crystal structure of complexes of inhibitors with the indicated proteins. Since the writing of the reviews by Khoury and Domling [4] and Popowicz, Domling and Holak [5], a significant number of new works have appeared. A feature of the present review is a more detailed description of synthetic approaches to known inhibitor compounds and the possibility of synthetic scheme changes, allowing the key reaction steps to be realized in an enantioselective version. Some attention is also given to the biotesting results and biomedical data of the obtained compounds.

The p53 protein, which is a tumor suppressor, is one of the potential targets of antitumor therapy. The role of this protein in living organisms is very large, and includes participation in the processes of DNA repair, cell cycle arrest, apoptosis, and aging; in this regard, it has received the name “guardian of the genome.” In more than 50% of tumor cell cultures, the p53 protein is mutated [6], which makes it possible to use a fairly wide range of biological and chemical methods for its activation or restoration of its function.

Activation of the p53 protein leads to one of two functions: initiating apoptosis or arresting cell growth [7]. p53-induced apoptosis occurs via the mitochondrial pathway through transcription-dependent or transcription-independent mechanisms and by the death receptor pathway through the transcriptional activation of FAS (a membrane dound proteine) and KILLER/DR5 (dead receptor) [8]. p53 is also able to transcriptionally suppress cell survival genes such as Bcl-2 (B-cell lymphoma 2), survivin, IGFR (insulin-like growth factor 1), Mcl-1 (induced myeloid leukemia cell differentiation protein), and PIK3CA (phosphatidylinositol-4,5-bisphosphate 3-kinase), through various mechanisms [9]. On the other hand, p53-induced cell cycle arrest is mainly due to an increase in the concentration of proapoptotic proteins p21 (cyclin-dependent kinase inhibitor 1), Gadd45 (Growth arrest and DNA-damage-inducible protein), 14-3-3σ, and PTGFβ, by direct binding to DNA or its transactivation [10]. Other p53-dependent anti-cancer mechanisms include the activation of accelerated cell aging [11], inhibition of angiogenesis [12], and regulation of autophagy [13].

p53 [14,15] triggers the expression of proteins with the following functions: (a) the proteins involved in control of the cell cycle; (b) the proteins involved in DNA repair processes; (c) the proteins that prevent angiogenesis; (d) the proteins with antioxidant action; (e) the proteins that regulate metabolism; and (f) inducers of apoptosis. The most important is the last group of proteins, which are capable of triggering various pathways of cell death. This group also includes the proapoptotic proteins Bax (also known as bcl-2-like protein 4), NOXA (phorbol-12-myristate-13-acetate-induced protein 1), and PUMA (p53 upregulated modulator of apoptosis) [14]. The main pathway of cell death controlled by the p53 protein is the mitochondrial apoptosis mechanism [16]. Proapoptotic proteins (Bax, NOXA, and PUMA), as well as the p53 protein itself, interact with the mitochondrial membrane, causing a decrease in its wall thickness. This leads to the release of cytochrome C from mitochondria into the cytoplasm. Cytochrome C interacts with proteins of the cell cytoplasm, triggering a cascade process for the activation of various caspases [17]. Caspases ruin the cytoskeleton, which leads to inevitable cell death. Therefore, there is a complex system of interactions between proteins and DNA that controls life processes and cell death.

In the occurrence of the cellular stress response, the level of p53 increases via the post-translational mechanism, which ultimately leads to cell cycle arrest or apoptosis. In the absence of cellular stress, the amount of p53 in the cell is controlled by a negative regulator of the p53 protein—the MDM2 protein. The MDM2 protein binds to p53 amino acid residues, causing ubiquitin-dependent p53 degradation [18]. The p53 and MDM2 proteins are linked to each other by a feedback mechanism, i.e., with an increase in the concentration of the p53 protein, a decrease in the concentration of the MDM2 protein is observed, and vice versa. Therefore, there is negative feedback between these proteins [14].

## 2. p53–MDM2 Inhibitors

Among the attempts to disrupt the regulation of the functioning of the p53-dependent mechanism are the following approaches: the synthesis of small-molecule inhibitors of the p53–MDM2 interaction, p53-dependent gene therapy, and the use of compounds that can bind to mutant p53 and restore its function.

### 2.1. Natural Compounds

About 60% of drugs on the market today (excluding biologics) have a natural origin [19,20]. To date, three natural-based compounds have been described as exhibiting inhibitory activity towards the p53–MDM2 interaction.

**Figure 1:**
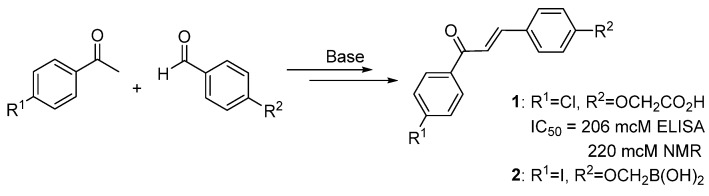
**1** and **2** as p53–MDM2 antagonists [21,22,23,24].

Chalcone-based inhibitors were the first reported compounds of this type and they have been the most extensively studied [21,22,23,24]. Chalcone **1** (Figure 1) had an ELISA IC_50_ value of 206 µM and caused a shift in the pattern of the ^1^H-^15^N HSQC NMR spectrum of ^15^N-enriched MDM2, consistent with chalcone binding in the tryptophan pocket. A gel shift assay revealed that the p53 released from the p53/MDM2 complex by treatment with **1** was unable to bind DNA, suggesting an additional influence of the chalcone on the p53 protein.

A series of boronic chalcone analogues were prepared by Khan and co-workers to address the lack of specificity in the carboxylic acid-containing chalcones [23]. They reported a modest improvement; compound **2** was 2.5- to 10-fold more toxic to human breast cancer cell lines than to a normal breast epithelial cell line at 10–40 µM. Isoliquiritigenin (4,2′,4′-trihydroxychalcone), which is a natural chalcone that is isolated from licorice root and shallot, has been shown to induce cell cycle arrest and apoptosis in liver cancer cells via the p53 pathway at 10–20 µg/mL, but its binding to MDM2 was not characterized [24].

**Figure 2:**
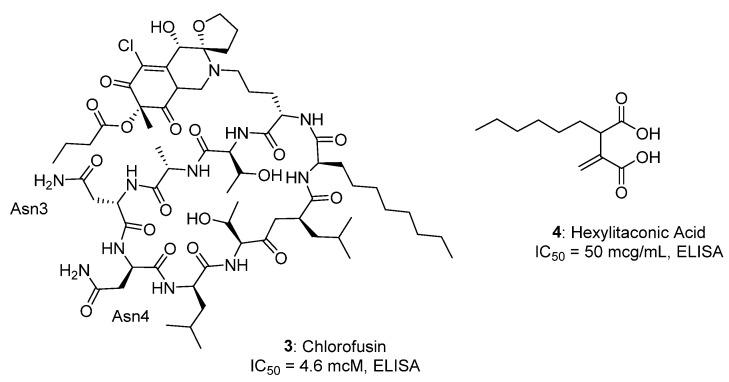
Structures of chlorofusin (**3)** and hexylitaconic acid (**4)** [25,26,27].

Chlorofusin was the second natural product inhibitor of p53/MDM2 to be reported [25]. Williams and co-workers identified chlorofusin as an inhibitor of p53/MDM2 binding (**3**, Figure 2) after testing over 53,000 extracts from the fermentation of a diverse collection of microorganisms for this activity [25]. This novel metabolite from the fungus *Microdochium caespitosum* had an IC_50_ of 4.6 µM in a p53/MDM2 ELISA. Further studies using SPR (surface plasmon resonance) confirmed that chlorofusin binds to the N-terminal region of MDM2 (Kd = 4.7 lM) [26].

Recently, another natural product inhibitor of the p53/MDM2 interaction, (-)-hexylitaconic acid (**4**, Figure 2), was reported [27]. Isolated from the fermentation culture of a *Arthrinium sp*. Fungus, which was isolated from a marine sponge, (-)-hexylitaconic acid had an IC_50_ of 50 µg/mL (~230 µM) for p53/MDM2. The inhibition of the p53–MDM2 interaction was tested by ELISA, according to the standard procedure, using purified recombinant p53 and HDM2 (human homologue of MDM2) proteins, and the following primary anti-MDM2 antibody. Other derivatives of **4**, including the monomethyl ester, a dihydro derivative, and a dihydro derivative of the monomethyl ester, as well as two commercially available dicarboxylic acids (itaconic acid and succinic acid) did not inhibit the interaction at all at the concentration of 50 μg/mL.

### 2.2. Nutlin Analogs

**Figure 3:**
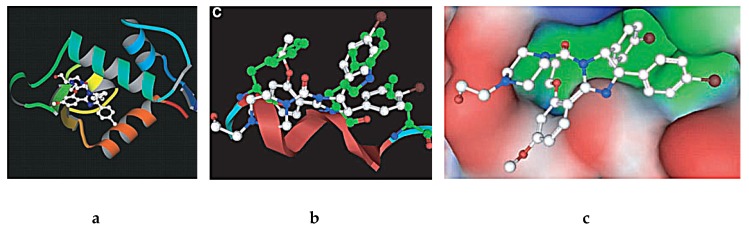
**(a)** MDM2 protein fragment with the Nutlin-3a inhibitor located at the p53 binding site. (**b**) Nutlin-3 overlay (carbon atoms are marked in white, nitrogen atoms in blue, oxygen atoms in red, and bromine in brown) and amino acid fragments of Phe19, Trp23, and Leu26 of the p53 protein. (**c**) The surface of the p53– MDM2 binding site (hollows are marked in green, and convex sections in red), showing one bromophenyl group located deep in the Trp pocket.

The most important push for the development of small-molecule inhibitors of the p53–MDM2 interaction was the development of 4,5-dihydroimidazoline (Nutlin). In 2004 [28], based on molecular modeling data, it was shown that the Nutlin-3 molecule is able to integrate into a small hydrophobic pocket of the MDM2 protein, simulating three amino acid residues in the p53 protein (Phe19, Trp23, and Leu26), which are the most important binding fragments. The crystal structure of one of Nutlin’s isomers (Nutlin-3a) in the first binding site to MDM2 is currently used as a model for creating new inhibitors of the p53–MDM2 protein–protein interaction [29] (Figure 3).

**Scheme 1:**
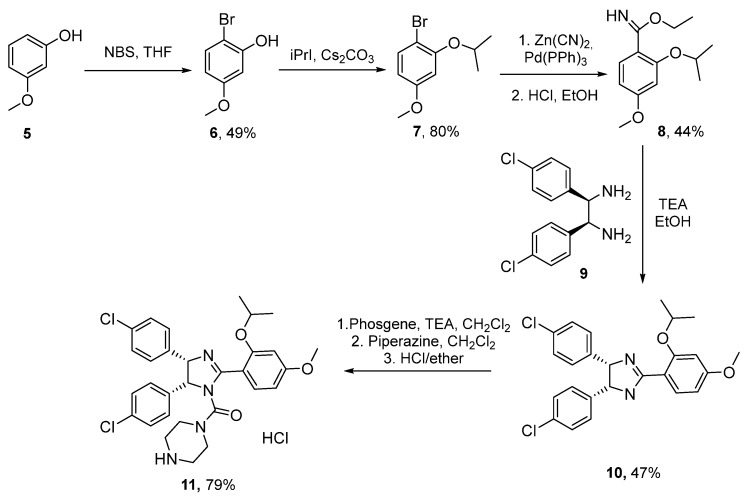
Nutlin-3 (**11**) synthesis as proposed by the pharmaceutical company Roche [30].

Nutlin-3 (Scheme 1, compound **11**), as a racemic mixture, demonstrates a cytotoxicity value on p53-expressing cell lines, with an IC_50_ value of about of 100–300 nm [4]. The enantiomers were separated on a chiral column, and when studying enantiomerically pure preparations, it was shown that (-)-Nutlin-3 (also called Nutlin-3a) is a 150 times more effective inhibitor compared to (+)-Nutlin-3. The synthesis of Nutlin by the pharmaceutical company Roche includes eight stages, with separation on a chiral chromatographic column (Scheme 1): initial bromination of 3-methoxyphenol (**5**), subsequent alkylation (**6**) to obtain isopropyl ether (**7**), and palladium-catalyzed cross-coupling with the formation of imine (**8**), which then reacts with *meso*- (4-chlorophenyl)ethane-1,2-diamine (**9**) to form imidazoline (**10**). Compound **10** reacts with phosgene to give carbamoyl chloride, which is then sequentially treated with piperazine and a solution of hydrogen chloride in ether, resulting in racemic Nutlin 3 (**11**). The separation of the latter on a chiral chromatographic column yields the Nutlin-3a active enantiomer [30].

**Scheme 2:**
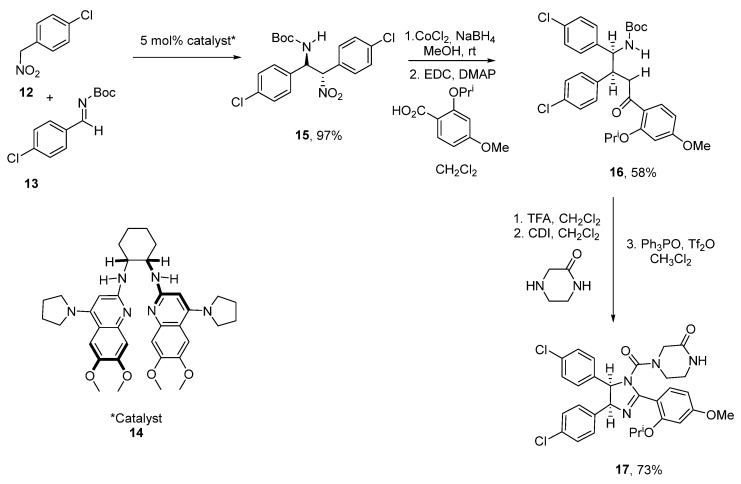
Nutlin-3a synthesis proposed by the researchers from Vanderbild University [31].

An alternative enantioselective method for Nutlin-3a synthesis, which includes only six stages (Scheme 2), was proposed by a group of researchers from Vanderbild University [31]. Initially, by diastereo- and enatioselective cross-coupling of a *para*-chloronitrobenzyl derivative **12** and the *Boc*-protected imine **13** in the presence of a chiral catalyst **14**, the nitro-substituted *cis*-stilbene **15** was obtained, which was reduced to amine using generated in situ cobalt boride; the amine was then acylated to obtain a *Boc*-protected amino amide **16**. After removal of the *Boc*-protection with trifluoroacetic acid, the resulting amine was acylated using carbonyldiimidazole, whereby an isocyanate was subsequently obtained, which was then treated with piperazinone and cyclized in the presence of triphenylphosphine oxide in Tf_2_O to form the desired Nutlin-3a. This method allowed the total number of stages to be reduced, and the stage of separation on a chiral column to be avoided.

**Scheme 3:**
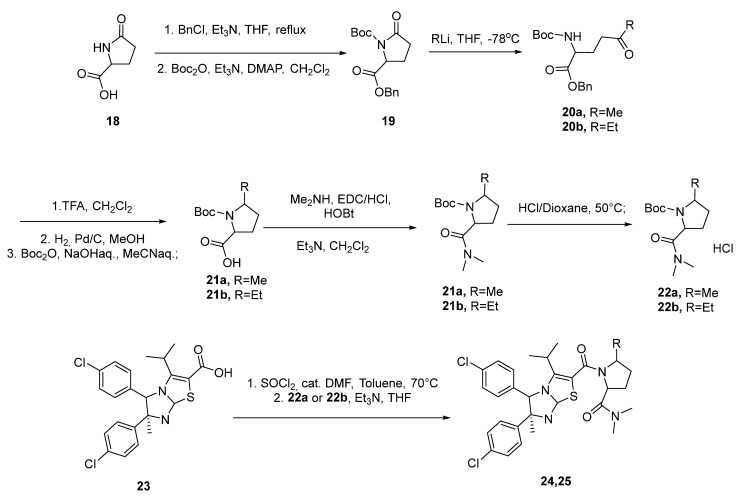
Pyrrolidine-based inhibitors synthesized by Daiichi Sankyo [32].

Scientists from Daiichi Sankyo reported the synthesis of compounds **24** and **25** using proline as the starting material (Scheme 3) [32]. Firstly, the reaction with alkyl lithium was carried out with the previous protection of amide and carboxylic groups. Then, the racemic pyrrolidine **22** was obtained in three stages.

Compound **24** (Protein Data Bank ID: 3W69), with an IC_50_ value of 59 nm (homogeneous time resolved fluorescence), also exhibited a good pharmacokinetic profile and significant antitumor efficacy via oral administration in a mouse xenograft model using MV4-11 cells bearing wild type (WT) p53.

**Scheme 4:**
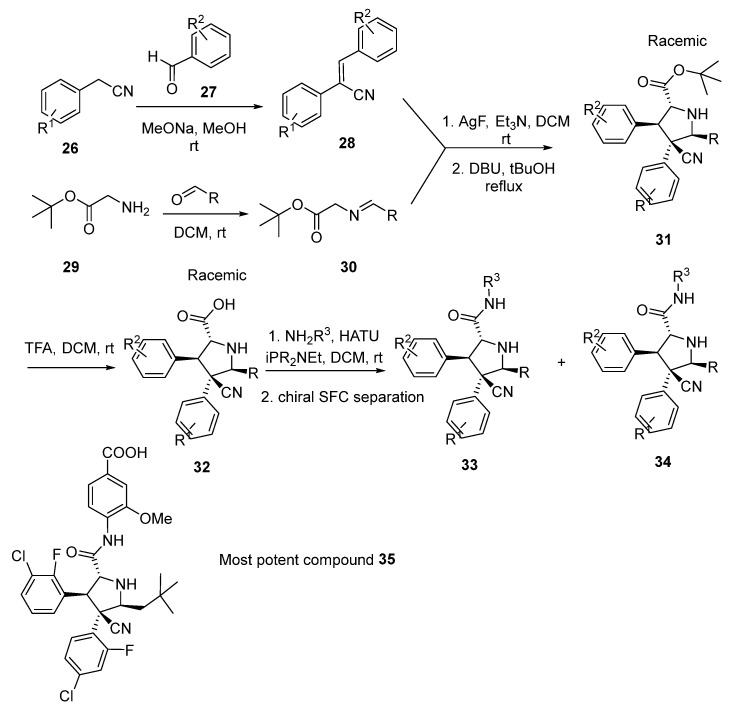
Synthesis of RG 7388, a potent and selective p53−MDM2 inhibitor in clinical development [34,35].

On the basis of the Nutlin-3a compound, the pyrrolidine-containing compound **32** was synthesized [33]. Starting from the condensation of benzyl cyanide derivatives **26** with aromatic aldehydes **27** in the presence of sodium methylate, and further imine addition, the racemic pyrrolidine derivative **31** was obtained. After *Boc*-deprotection by triflic acid and amine addition, the racemic amide **32** was formed and then separated by a chiral supercritical fluid chromatography (). The most potent compound **35** is shown in Scheme 4 [34,35].

**Scheme 5:**
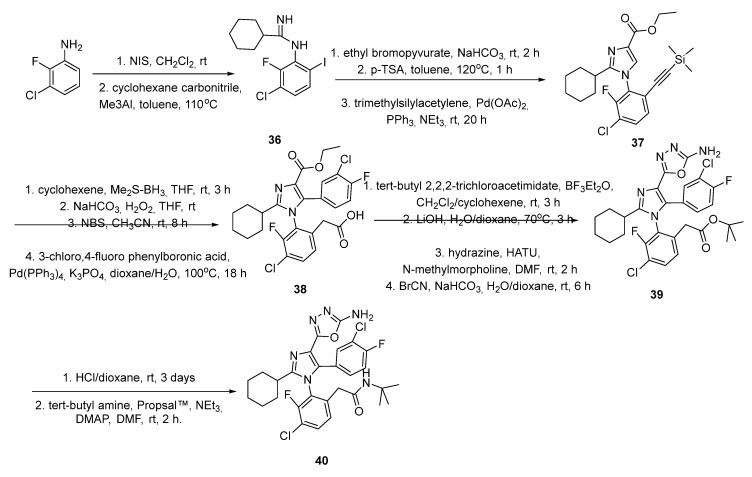
Syntheses of inhibitor **40** [36].

In 2014, Furet and co-workers published a new class of tetra-substituted imidazoles as a new class of inhibitors of the p53–MDM2 interaction (Scheme 5) [36]. Commercially available 2-fluoro-3-chloro-aniline was subjected to iodination with N-Iodosuccinimide (NIS), providing a regioisomeric mixture of 4- and 6-iodo products, which were separable by flash chromatography. The desired 6-iodo regioisomer was reacted with cyclohexane carbonitrile in the presence of trimethyl aluminum to form the corresponding benzamidine **36**, which underwent smooth cyclization with ethylbromo-pyruvate under mild basic conditions (NaHCO_3_; room temperature). Water elimination was effected by the addition of p-toluene sulfonic acid and heating to 120 °C to furnish the imidazole core. Selective Sonogashira coupling of the iodine with trimethyl silyl acetylene provided intermediate **37**. Conversion of the acetylene side chain to the desired acid was achieved by hydroboration (cyclohexene/borane-dimethylsulfide complex) and oxidative workup. Efficient and selective bromination of the imidazole core was effected by treatment with N-Bromosuccinimide (NBS) in acetonitrile at room temperature, providing the suitable substrate for Suzuki coupling with commercially available 3-chloro-4-fluoro boronic acid. Orthogonal ester protection/deprotection steps provided acid **38**, which was converted to the 2-amino-oxadiazole in a two-step sequence (HATU ((1-[Bis(dimethylamino)methylene]-1H-1,2,3-triazolo[4,5-b]pyridinium 3-oxide hexafluorophosphate, **H**exafluorophosphate **A**zabenzotriazole **T**etramethy **U**ronium) promoted hydrazone formation and ring closure with BrCN). Finally, deprotection of the *tert*-butyl ester **39** liberated carboxylic acid, which was converted to the corresponding carboxamide **40** using Propsal™ as a coupling reagent.

The most potent compounds of the series show significant and specific anti-proliferative activity in cultures of p53-dependent cancer cells. These results warrant further evaluations of the new inhibitors towards the goal of developing anti-cancer agents to fight tumors harboring an overexpressed or amplified MDM2 gene [36].

### 2.3. Spirooxindole Derivatives

**Scheme 6:**
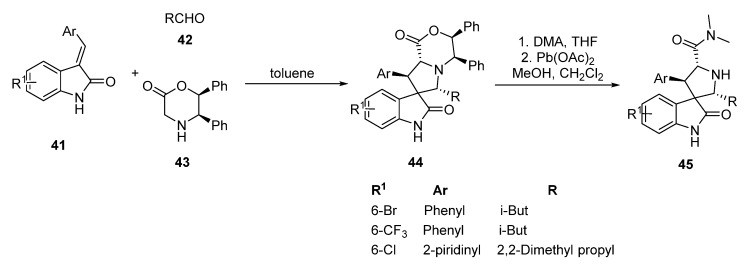
The general scheme for the synthesis of compounds of the MI series, proposed by a group of researchers from the University of Michigan [38].

The most promising MDM2 inhibitor sare possibly the compounds obtained by Shaomeng Wang’s group from the University of Michigan [37]. Based on molecular docking data for these structures, the group proposed modeling the tryptophan fragment in the p53 protein with a spirooxindole fragment, which, due to its greater conformational rigidity, is able to provide a better affinity for the MDM2 protein. This class of compounds can be obtained as shown in Scheme 6, with high stereoselectivity. The condensation reaction of the aromatic aldehydes with oxindoles was initially carried out either in the presence of a base during boiling or using microwave radiation, to obtain *E*-3-aryl-1,3-dihydroindol-2-ones **41**, which were further introduced into 1,3-dipolar cycloaddition reaction with aliphatic aldehydes **42** and optically active (*5R,6S*) -5,6-diphenylmorpholin-2-one **43** in toluene. The resulting product **44** was purified by column chromatography and then treated with a solution of dimethylamine in tetrahydrofuran and lead(IV) acetate to give amide **45** in a quantitative yield. The absolute configuration of the products was confirmed by X-ray diffraction data [38].

**Figure 4:**
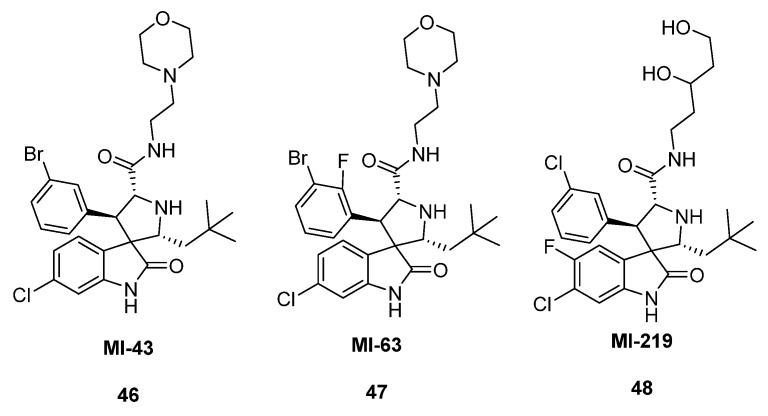
Most active compound-leaders of the MI series, synthesized by a team of researchers from the University of Michigan [39,40].

The synthesized compounds showed a significant cytotoxic effect on the prostate cancer cell line LNCap, with IC_50_ = 86 nM, and on the colorectal cancer cell line HCTwt, with IC_50_ = 22 μM. Based on this method, a large library of spiro derivatives was synthesized, the most active of which are shown in Figure 4. Compounds MI-43 (**46**), MI-63 (**47**), and MI-219 (**48**) showed excellent binding to the MDM2 protein (Ki~5.7 nM), and compound MI-219 (**48**) was recognized as a selective inhibitor of the p53–MDM2 interaction due to its ability to induce cell apoptosis in tumor cells without affecting healthy ones [39]. Upon an oral injection of compound **48** in xenograft models with the SJSA-1 grafted tumor line, the inhibition of tumor growth was 86% at a double dose of 200 mg/kg [40].

**Figure 5:**
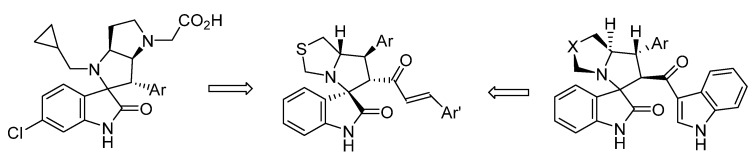
Spiroindolinones design in the work [41].

In 2019, Barakata and co-workers [41] synthesized a series of spiroindolinones using an asymmetric 1,3-dipolar reaction as the key step (Figure 5). Their investigation involved the design and synthesis of substituted spirooxindoles as potent MDM2 inhibitors, using an efficient 1,3-dipolar cycloaddition reaction [42,43]. The one-pot multi-component reactions of a α,β-unsaturated dienone derivative with substituted isatines and amino acid derivative (*l*-4-thiazolidinecarboxylic acid), and underheating in MeOH at 60 °C for 1.5–2.0 h, were conducted to generate the focused cycloadducts library, which had four stereogeneric centers with an excellent yield (69–89%).

The anticancer activities of the synthesized compounds were tested against colon (HCT-116), prostate (PC-3), and hepatocellular (HepG-2) cancer cell lines. The mechanism underlying the anti-cancer activity of the obtained spiroindolinones was further evaluated, and the study showed that these compounds inhibited colony formation and cell migration, arrested cancer cell growth at G2/M, and induced apoptosis through intrinsic and extrinsic pathways. The transactivation of p53 was confirmed using flow cytometry, where tested compounds increased the level of activated p53 and induced mRNA levels of cell cycle inhibitor p21.

**Scheme 7:**
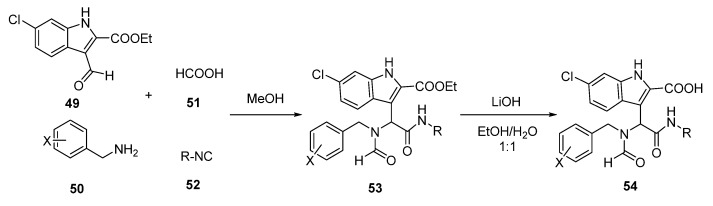
General scheme for the synthesis of inhibitors of the p53–MDM2 interaction obtained by the Ugi reaction [44].

A series of compounds using the four-component Ugi reaction (Scheme 7) [44] were obtained. The four-component Ugi reaction was carried out by mixing equivalent amounts of ethyl 6-chloroindole-2-carboxylate **49**, benzylamine **50**, formic acid **51**, and the corresponding isocyanide **52** in methanol. The resulting esters **53** were hydrolyzed to give the acid **54**.

Using molecular docking results, it was found that in the co-crystalline structure of molecule **54** with the MDM2 protein, there is a π–π-stacking interaction between the benzylamine fragment in the inhibitor molecule and the His96 protein fragment. Based on these data, a library of 19 possible isomers of compound **54**, with different positions of the fluorine atom in the benzyl position, was obtained [44]. These compounds were tested for binding to the MDM2 protein in the form of racemic mixtures; however, only one of the synthesized molecules with a 3,4,5-trifluorobenzylamine substituent was active and showed a binding constant of 130 nM^−1^.

**Scheme 8:**
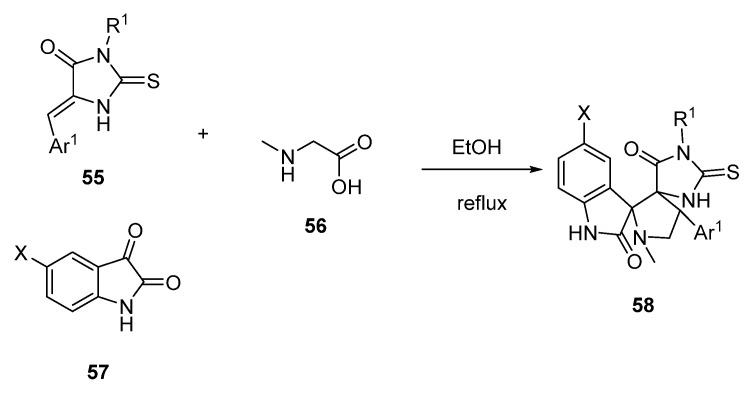
General scheme for the synthesis of dispiroindolinone **58** [45,46].

In 2014, the dispiro-compound **58**, as a potential p53–MDM2 inhibitor, was proposed [45,46]. This compound was obtained via a 1,3-dipolar cycloaddition reaction with isatine **57**, sarcosine **56**, and thiohydantoin-based dipolarophiles **55** (Scheme 8).

The most potent compound had IC_50_ =1.08 ± 0.96 µM on LNCap cell lines.

### 2.4. Benzodiazepine-2,5-diones (BDP)

**Scheme 9:**
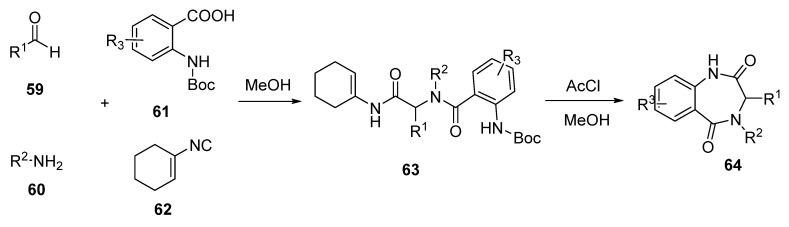
General scheme for the synthesis of compounds of the benzodiazepine (BDP) series [47].

The pharmaceutical company Johnson and Johnson screened a library of 1,4-benzodiazepine-2,5-dione (BDP) capable of binding to the p53 domain of the MDM2 protein. These compounds can be obtained in two stages (Scheme 9), with the initial multi-component Ugi reaction between equimolar amounts of aldehyde **59**, amine **60**, *Boc*-protected anthranilic acid **61**, and 1-isocyanidecyclohexane **62** in methanol, to obtain the product **63**, which then forms target cyclic product **64** under the action of acetyl chloride [47]. The most active compound of this group is the enantiomer containing the *para*-chlorophenyl group at R^1^, an α-carboxybenzyl group at R^2^, and an iodine atom at R^3^; this compound has a binding constant with the MDM2 protein of K_d_ = 80 nM. 

**Scheme 10:**
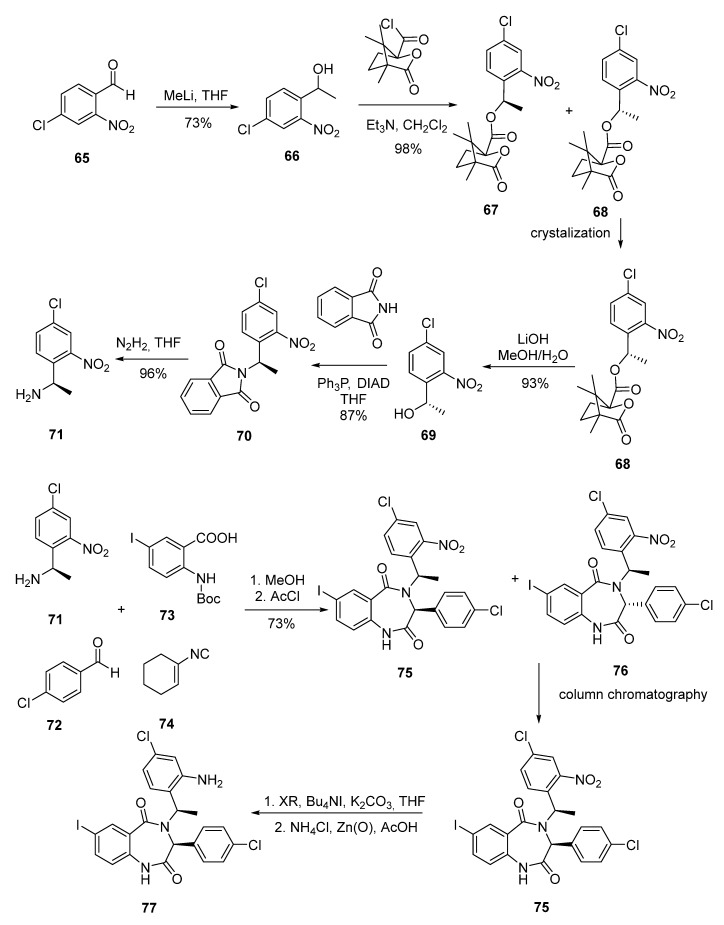
General scheme of the enantioselective pathway for the synthesis of compounds of the BDP series [48] (DIAD is diisopropyl azodicarboxylate).

In the next investigation step, Johnson and Johnson undertook an enantioselective synthesis of the benzodiazepine compounds of the BDP series (Scheme 10). Compound **65** was initially introduced into an alkylation reaction with methyl lithium to form a racemic secondary alcohol **66**, which reacted with camphoric acid chloride to form a mixture of two diastereomers. This mixture was then recrystallized to isolate ester **68** as a single stereoisomer, which was then hydrolyzed and introduced into a Mitsunobu reaction with phthalimide, and the phthalimide protection was then removed with hydrazine to form amine **70**. This amine was used in the Ugi reaction, followed by cyclization, described above, and the mixture of diastereomers was then separated by column chromatography, after which alkylation at the nitrogen atom yielded the necessary compound **71 [48]**, the structure of which was confirmed by X-ray diffraction.

Compound **71** was further modified by introducing carboxyalkyl groups at the unsubstituted amide nitrogen atom, as well as by replacing the nitro group with an amino group. The obtained compound showed higher than initial activity in a polarization fluorescence immunoassay (0.25 µM compared to 0.85 µM), since the presence of the amino group ensured binding to the Val93 carbonyl atom in the MDM2 protein, but it showed less activity in cytotoxicity experiments, possibly due to its zwitterionic structure, which limited the ability to penetrate the cell membrane. The authors also made attempts to introduce morpholine and isopropyloxy groups into the target molecule; however, these substitutions led to a decrease in cytotoxicity and loss of activity.

A group of scientists led by Alexander Domling of the University of Pittsburgh, together with Thad Holak of the Max Planck Institute, also proposed an interesting approach to creating p53–MDM2 interaction inhibitors using the multi-component Ugi reaction [49,50].

### 2.5. Chromenotriazole Pyrimidines

**Scheme 11:**
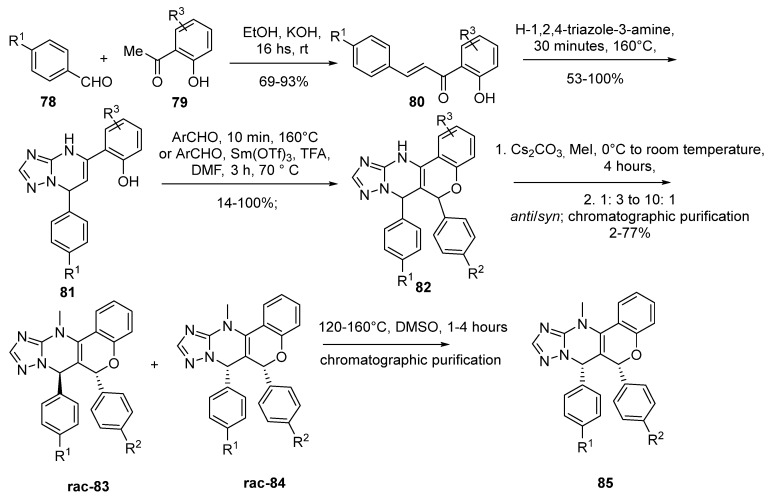
General scheme for the synthesis of inhibitors of the p53–MDM2 interaction based on chromenotriazolepyrimidines [51].

Another interesting example of the development of p53–MDM2 interaction inhibitors is the synthesis of chromenotriazole pyrimidine derivatives (Scheme 11) [51]. Initial aldol condensation of aldehydes **78** and methyl ketones **79** in an alkaline medium affords hydroxychalcones **80**, which then condensate with 4H-1,2,4-triazole-3-amine by heating to give compounds **81** as the mixtures of two tautomers. The desired enamine tautomer can be isolated by mashing in chloroform. The condensation of compound **81** with various aldehydes by heating or in an acidic medium with phenols allows the condensation products **82** to be obtained as mixtures of stereoisomers. After the methylation of compounds **82** at the nitrogen atom, 11-methylchromenotriazolepyrimidines **83** and **84** were formed as the mixture of the racemic *anti*- and *syn*-isomers, which could be separated by column chromatography, giving the target compound **85**.

The best cytotoxicity values in the analysis using the method of homogeneous fluorescence with time resolution (HTRF assay) showed the compound with R^1^ = Br and R^2^ = Cl (IC_50_ = 0.89 ± 0.20 μM). 

### 2.6. Piperidinones

**Scheme 12:**
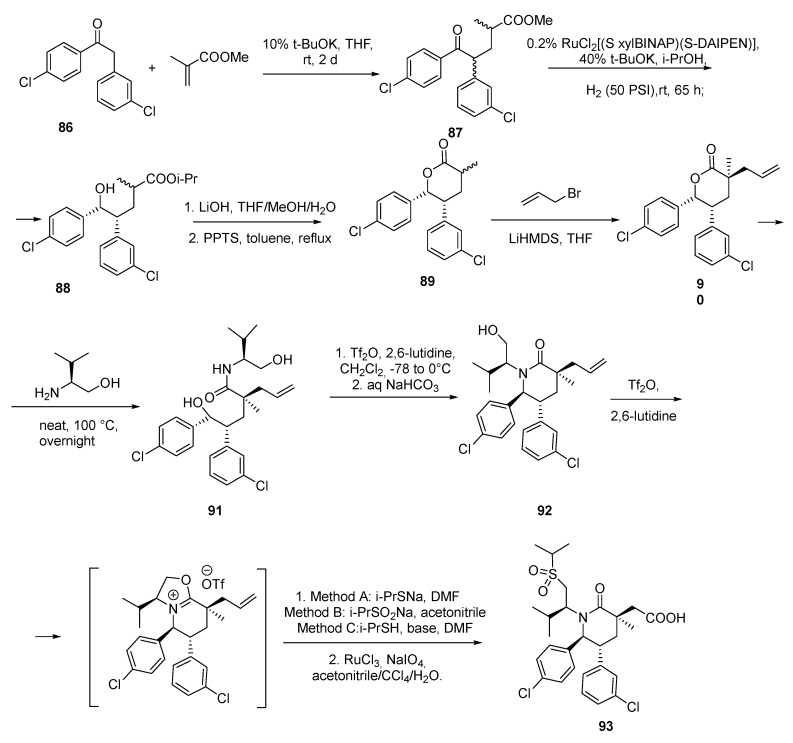
The synthesis of piperidinone **93** [52].

A piperidinone compound AMG 232 (**93**) was synthesized using the Michael condensation of ketone **86** and methacrylic acid ester, to get compound **87**. Then, reduction and ester formation with further cyclization at base conditions allowed cyclic lactone **89** to be obtained. The subsequent alkylation with allyl bromide and the reaction of the lactone opening by an amine derivative gave the chiral amide **91**, which was then cyclized to epypiperidinone **92** (Scheme 12) [52,53].

**Scheme 13:**
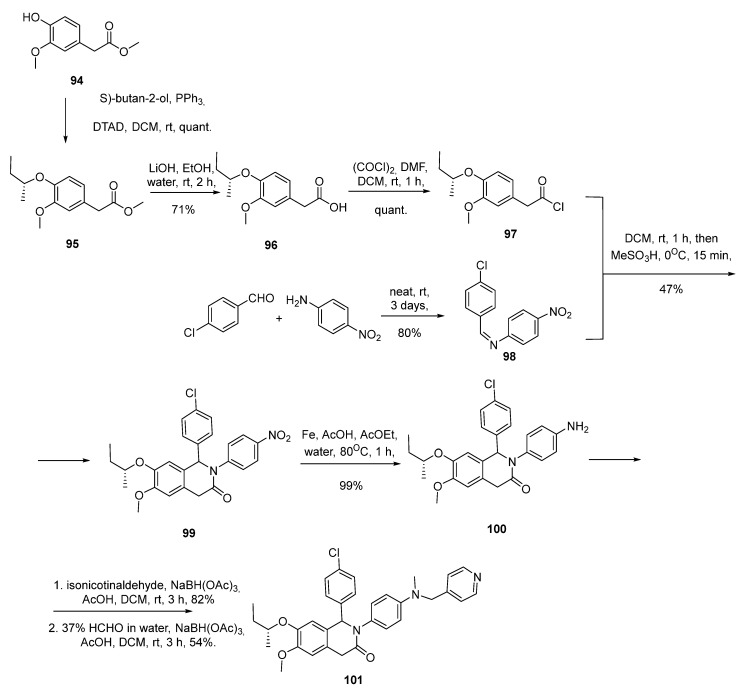
The synthesis of piperidinone **101** [54].

In 2015, Gessier and co-workers published a synthesis of a new class of the piperidinone-base small-molecule inhibitors of the p53–MDM2 protein–protein interaction (Scheme 13). Starting from an initial hit identified by virtual screening, a derivatization program resulted in compound **101**, which is a low nanomolar inhibitor of the p53–MDM2 interaction showing significant cellular activity [54,55]. This compound reached a low nanomolar biochemical potency, accompanied by significant and specific inhibition of the proliferation of the p53-dependent SJSA-1 cells in the low micromolar range. The production of compound **101** allowed an interesting level of cellular potency to be reached, which was comparable to that of the reference p53–MDM2 inhibitor Nutlin-3a (IC_50_ = 1.9 µM in the SJSA-1 assay).

A representative synthesis for compound **101** is described in Scheme 13. The commercially available methyl 2-(4-hydroxy-3-methoxyphenyl)acetate was subjected to a Mitsunobu reaction with (*S*)-butan-2-ol, using diethyl azodicarboxilate (DEAD) and triphenylphosphine as the reagents, to deliver the sec-butyl ether **95** as its pure (*R*)-enantiomer. The methyl ester **95** was hydrolyzed with LiOH into the corresponding carboxylic acid **96**, and then reacted with oxalyl chloride to give the acyl chloride **97** in quantitative yields. The cycloaddition reaction of **97** with the imine **98**, obtained from the condensation of the commercially available 4-chlorobenzaldehyde and 4-nitroaniline, led to the dihydroisoquinolinone analog **99**, with no stereo selectivity observed for the newly created asymmetric center. The nitro group of **99** was reduced with iron following classical reaction conditions, to give the corresponding aniline **100** in nearly quantitative yields. Finally, the aniline functionality underwent two consecutive reductive aminations with isonicotinaldehyde and formaldehyde, respectively, using NaBH(Oac)_3_ as a reducing agent, to deliver compound **101** as a mixture of two diastereoisomers. At that point, the separation of the diastereoisomeric pair could not be achieved by classical normal phase or reversed phase chromatography and required the use of chiral chromatography conditions.

Therefore, this potent inhibitor has been the starting point of another round of medicinal chemistry optimization that has culminated in NVP-CGM097 (highly potent and selective MDM2 inhibitor), which is a compound currently under evaluation in a phase I clinical trial for cancer patients [54,55].

### 2.7. Peptide-Based Compounds

**Figure 6:**
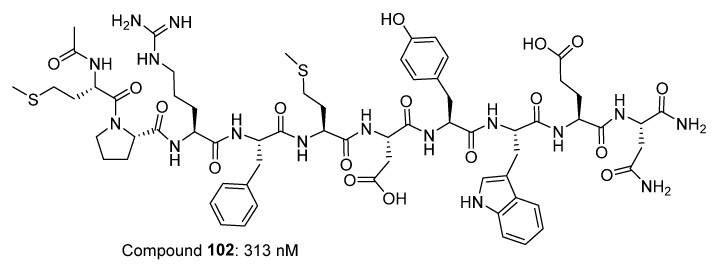
Peptide **102** [57].

Peptides and peptide derivatives can be designed to become potent p53–MDM2/X interaction inhibitors. The Novartis group mapped the MDM2-binding site on p53 using synthetic peptide libraries derived from the N-terminal region of p53. The active peptides defined the consensus MDM2-binding site on p53 to be Thr18-Phe-Ser-Asp-Leu-Trp23. This hexapeptide, however, was only a weak inhibitor, with an IC_50_ value, or concentration required for the inhibition of 50% of p53 binding to MDM2, of 700 µM in an ELISA format [60]. The most active peptide obtained in this way (Figure 6) showed a 28-fold greater inhibition of the p53/MDM2 interaction than the wild-type p53-derived peptide. (Peptide **102** was effective at inhibiting the p53/MDM2 interaction in cells [56]; in addition, this sequence was active when expressed either with a glutathione S-transferase tag51 or in the active-site loop of thioredoxin [57].

## 3. Conclusions

Fifteen years after the discovery of Nutlin, a number of new potential inhibitors of the p53–MDM2 interaction have been proposed, some of which have shown a high cytotoxicity and encouraging results in preclinical and clinical studies. Over the past decade, some attention has been paid both to the search for new small molecules and the improvement of the biomedical properties of already known classes of compounds, as well as to the modification of existing synthesis schemes to obtain enantiomerically pure compounds. Further directions of research in this area will depend on the results of ongoing clinical trials. Among the most promising classes of small molecules tested for the inhibition of p53–MDM2 protein–protein interactions are Nutlin’s analogues, as well as spirooxindoles and benzodiazepine-2,5-diones, whose rigid polycyclic framework allows one to achieve the optimal arrangement of substituents in the molecule necessary for interactions with the MDM2 binding site.
